# Effects of Preoperative HbA1c Levels on the Postoperative Outcomes of Coronary Artery Disease Surgical Treatment in Patients with Diabetes Mellitus and Nondiabetic Patients: A Systematic Review and Meta-Analysis

**DOI:** 10.1155/2020/3547491

**Published:** 2020-02-28

**Authors:** Jinjing Wang, Xufei Luo, Xinye Jin, Meng Lv, Xueqiong Li, Jingtao Dou, Jing Zeng, Ping An, Yaolong Chen, Kang Chen, Yiming Mu

**Affiliations:** ^1^Department of Endocrinology, Chinese PLA General Hospital, Beijing 100853, China; ^2^Departmentof Endocrinology, South Hospital District, Fifth Medical Center of PLA General Hospital, Beijing 100071, China; ^3^School of Public Health, Lanzhou University, Lanzhou 730000, China; ^4^Evidence-Based Medicine Center, Basic Medical Sciences, Lanzhou University, Lanzhou 730000, China; ^5^Key Laboratory of Evidence Based Medicine and Knowledge Translation of Gansu Province, Lanzhou University, Lanzhou 730000, China; ^6^WHO Collaborating Centre for Guideline Implementation and Knowledge Translation, Lanzhou 730000, China; ^7^Department of Gerontology, First Affiliated Hospital of Kunming Medical University, Kunming 650031, China

## Abstract

**Aims:**

To investigate the effect of preoperative HbA1c levels on the postoperative outcomes of coronary artery disease surgery in diabetic and nondiabetic patients.

**Methods and Results:**

The MEDLINE (via PubMed), Cochrane Library, Web of Science, Embase, Wanfang Data, China National Knowledge Infrastructure (CNKI), and Chinese Biology Medicine (CBM) databases were used to search the effects of different preoperative HbA1c levels on the postoperative outcomes of coronary artery disease surgical treatment in diabetic and nondiabetic patients from inception to December 2018. Two review authors worked in an independent and duplicate manner to select eligible studies, extract data, and assess the risk of bias of the included studies. We used a meta-analysis to synthesize data and analyze subgroups, sensitivity, and publication bias as well as the GRADE methodology if appropriate. The literature search retrieved 886 records initially, and 23 cohort studies were included in the meta-analysis. In this meta-analysis, we found that there was a reduced incidence of surgical site infections (OR = 2.94, 95% CI 2.18-3.98), renal failure events (OR = 1.63, 95% CI 1.13-2.33), and myocardial infarction events (OR = 1.69, 95% CI 1.16-2.47), as well as a shortened hospital stay (MD = 1.08, 95% CI 0.46-1.71), in diabetic patients after coronary artery disease surgical treatment with lower preoperative HbA1c levels. For nondiabetic patients, a higher preoperative HbA1c level resulted in an increase in the incidence of mortality (OR = 2.23, 95% CI 1.01-4.90) and renal failure (OR = 2.33, 95% CI 1.32-4.12). No significant difference was found between higher and lower preoperative HbA1c levels in the incidence of mortality (OR = 1.06, 95% CI 0.88-1.26), stroke (OR = 1.49, 95% CI 0.94-2.37), or atrial fibrillation (OR = 0.94, 95% CI 0.67-1.33); the length of ICU stay (MD = 0.20, 95% CI -0.14-0.55); or sepsis incidence (OR = 2.49, 95% CI 0.99-6.25) for diabetic patients or for myocardial infarction events (OR = 1.32, 95% CI 0.27-6.31) or atrial fibrillation events (OR = 0.99, 95% CI 0.74-1.33) for nondiabetic patients. The certainty of evidence was judged to be moderate or low.

**Conclusion:**

This meta-analysis showed that higher preoperative HbA1c levels may potentially increase the risk of surgical site infections, renal failure, and myocardial infarction and reduce the length of hospital stay in diabetic subjects after coronary artery disease surgical treatment and increase the risk of mortality and renal failure in nondiabetic patients. However, there was great inconsistency in defining higher preoperative HbA1c levels in the studies included; we still need high-quality RCTs with a sufficiently large sample size to further investigate this issue in the future. This trial is registered with CRD42019121531.

## 1. Introduction

Diabetes mellitus (DM) is a chronic disease with dysfunction in insulin secretion that gradually affects critical organs and tissues, including the heart, nerves, blood vessels, and eyes, and increases the risk of their infection [1]. The International Diabetes Federation reported that 415 million people, i.e., 8.8% of the world population, suffered from DM in 2015 [2]. Among them, 75% were from low- and middle-income countries. China, with 109.6 million people suffering from diabetes, ranked number one in the world.

With a sharply rising trend, the number of global diabetic patients and the global prevalence rate are estimated to reach 642 million and 10.4%, respectively, in 2040. At that time, the number of diabetic patients in China will reach 150.7 million. In 2015, DM resulted in approximately five million deaths in the world. Spending on the treatment of DM was estimated to be between 673 and 1197 billon US dollars, which was approximately 11.6% of the total world spending on health. As one of the most important noninfectious diseases, diabetes brings about a heavy burden on patients and their families, societies, and countries in terms of health and economic outcomes.

With the increase in diabetes prevalence, an increasing number of diabetic patients will have surgical operations. Statistics show that approximately half of DM patients have the chance to receive at least one surgery in life [3]. Current evidence indicates that perioperative blood glucose abnormalities (including hyperglycemia, hypoglycemia, and blood glucose fluctuation) could increase the rate of death and the incidence of complications such as infections, poor wound healing, and cardio/cerebrovascular events; prolong the duration of hospitalization; and affect long-term prognosis [4, 5]. For diabetic patients undergoing cardiac surgery, hyperglycemia could increase mortality, cause adverse events in the kidney and lung, increase the incidence of atrial fibrillation, and even lead to a serious threat to life [6–8]. Therefore, diabetic patients who undergo surgical treatments, especially those undergoing heart surgery, need strict management of blood glucose.

Glycosylated hemoglobin A1c (HbA1c) is usually regarded as a marker for the average blood glucose level over the three months before the measurement and is used for assessing glycemic control. Currently, many guidelines have provided recommendations on the preoperative HbA1c level for achieving better surgical outcomes, but the levels vary significantly. Some guidelines recommended that the HbA1c level should be below 7% or 6.5% [9, 10], but others recommended a level at 8.5% or 8%-9% [11, 12]. A systematic review [13] indicated that elevated preoperative HbA1c levels did not increase the morbidity and mortality of operations (including cardiac surgery) in diabetic patients; however, it did not mention the high threshold of preoperative HbA1c explicitly, which is not suitable to support the formulation of a recommendation. For nondiabetic patients, the relationship between outcomes after cardiac surgery and preoperative HbA1c level is still unclear.

Our study focused on the impact of preoperative HbA1c levels on the postoperative outcomes of coronary artery disease surgical treatment in diabetic and nondiabetic patients, and we conducted a meta-analysis by retrieving and reviewing systematically relevant research evidence. In addition, this study will provide guidance for clinical practice by objectively presenting the results with the GRADE system.

## 2. Materials and Methods

### 2.1. Search Methods

A number of database resources, including MEDLINE (via PubMed), Embase, the Cochrane Library, Web of Science, Wanfang, CNKI, and CBM, were searched from the inception of each resource to December 12, 2018. The WHO International Clinical Trials Registry Platform (ICPTR) and ClinicalTrials.gov were searched as supplements without limits to the publication types. The following keywords were used: diabetes, cardiac surgery, and preoperative. We also searched references from original articles, clinical guidelines, narrative reviews, and previous systematic reviews/meta-analyses to identify additional studies. We followed the PRISMA guidelines for conducting and reporting meta-analyses of RCTs (Supplementary [Supplementary-material supplementary-material-1]) [14]. [Supplementary-material supplementary-material-1] detailed the search strategy.

### 2.2. Eligibility Criteria

#### 2.2.1. Population

Diabetic patients who met the diagnostic criteria for DM of the American Diabetes Association [15] and who underwent elective cardiac surgery were eligible for inclusion in the study. We mainly focused on coronary artery bypass grafting (CABG) and percutaneous coronary intervention (PCI) and ignored the other risk factors that most likely affect the outcomes of elective cardiac surgery, such as cardiovascular risk factors and medical history. We also referred to the indications for adult cardiac surgery created by Young [16]. In addition, we included nondiabetic patients who also underwent an elective cardiac surgery to compare with the diabetic patients.

#### 2.2.2. Exposure Factors/Controlled Factors

We mainly included diabetic and nondiabetic patients whose preoperative HbA1c cut-off value was 6.5% and 7%, respectively. We defined higher HbA1c levels as preoperative HbA1c ≥ 6.5% or 7% and lower HbA1c levels as preoperative HbA1c < 6.5% or 7%.

#### 2.2.3. Outcomes

We analyzed perioperative health-related outcomes. The primary outcome was mortality. The secondary outcomes were hospital stay, the length of ICU (intensive care unit) stay, cardiovascular events (such as atrial fibrillation and stroke), and other adverse events (such as renal failure and sepsis).

#### 2.2.4. Types of Studies

Prospective randomized controlled trials (RCTs), cohort studies, and retrospective studies were included in this meta-analysis.

#### 2.2.5. Selection of Studies

Two reviewers independently screened titles, abstracts, and the full texts of the identified studies. There was a pretest and discussion before the formal screening of the literature to ensure the consistency between reviewers. Two reviewers solved any disagreement by discussion or consultation with a third researcher.

#### 2.2.6. Data Extraction

Data extraction was undertaken independently by two reviewers using standard data extraction templates with the following information: basic information (publication year, first author, institution, and journal) and contents of studies (study design, sample size, research objective, the characteristics of population, preoperative HbA1c levels, and health-related clinical outcomes). A pretest was performed before the formal extraction to ensure that each reviewer was consistent in their understanding of the criteria and process of extraction. Any disagreement was solved by discussion or consultation with a third reviewer. If any important data in the original study provided inadequate information, attempts would be made to acquire the necessary information by contacting the authors, and if we could not obtain a reply within one week, we excluded the study.

#### 2.2.7. Assessment of the Risk of Bias in the Included Studies

Two reviewers used the Cochrane bias risk assessment tools [17] to assess the included RCTs and used the Newcastle Ottawa Scale (NOS) [18] for cohort studies and retrospective studies. For the Cochrane bias risk assessment tools, the criteria we used included random sequence generation, allocation concealment, the blinding of participants and personnel, the blinding of outcome assessors, incomplete outcome data, selective reporting, and other biases. A judgment of “unclear” indicated an unclear or unknown risk of bias. We judged individual bias items individually for each study as described in the *Cochrane Handbook for Systematic Reviews of Interventions*. If a cohort study or retrospective study had an NOS score lower than seven, it indicated that there was a serious bias in this study. A pretest was performed before the formal assessment to ensure that each reviewer was consistent in their understanding of the criteria and process of evaluation. Any disagreement was solved by discussion or consultation with a third researcher.

#### 2.2.8. Statistical Analysis

The random-effect model was used for all analyses. Mean differences and 95% confidence intervals (CIs) were calculated for continuous outcomes. For dichotomous data, odds ratios and 95% CIs were calculated instead. Heterogeneity was identified by using the *Q* test (*P* < 0.05, suggesting the existence of heterogeneity). Heterogeneity was also specifically examined by employing the *I*^2^ statistic. Inconsistency across studies was determined to assess the impact of heterogeneity on the meta-analysis. An *I*^2^ statistic of 50% or more indicated a considerable level of inconsistency. The data were summarized statistically provided they were available, sufficiently similar, and of sufficient quality [19]. Statistical analyses were performed according to the guidelines referenced in the 2011 version of the *Cochrane Handbook* using RevMan 5.3 software. Sensitivity analysis was used to investigate the source of heterogeneity using Stata 14.0 software, provided that obvious or significant heterogeneity was observed. In addition, publication bias was performed if the included studies were more than nine.

#### 2.2.9. Subgroup Analysis

We predesigned the subgroups on the basis of different levels of preoperative HbA1c cut-off values and the type of operation (CABG and PCI).

#### 2.2.10. Grading of Quality of Evidence

GRADE (Grading of Recommendations Assessment, Development and Evaluation) [20–22] was used to assess the quality of evidence for each outcome. The criteria mainly considered included the risk of bias, indirectness, inconsistency, imprecision, and publication bias. The quality of evidence for each outcome was graded as high, moderate, low, or very low. Finally, we presented the results of the quality of evidence for each outcome in a table that summarizes the findings.

## 3. Results

### 3.1. Results of the Search

**Figure 1:**
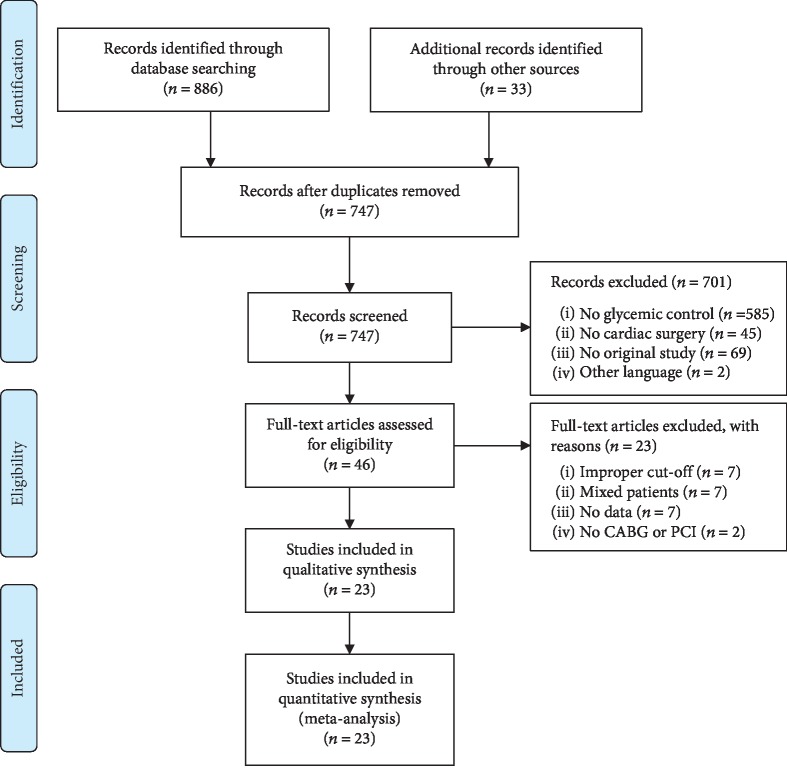
Flowchart of the study selection.

The literature search retrieved 886 records preliminarily, and 701 studies were excluded by screening the title and abstract. After the removal of 46 studies for not meeting the inclusion criteria, 23 studies were eligible for inclusion [23–45].  Figure 1 shows the flowchart of study selection.

### 3.2. Baseline Characteristics of Included Studies

Twenty-three studies were included.  Table 1 details the baseline characteristics of the included studies.

### 3.3. Risk of Bias

Most of the included studies did not report on the independent blind assessment of outcomes. In terms of follow-up, 9 studies [23, 24, 26, 28, 31, 36, 37, 42, 43] did not report follow-up time and one study [41] had a 30-day follow-up, which was relatively short for outcomes and had high bias. Other studies were at low risk of bias in other items (see Table 2).

### 3.4. Mortality

**Figure 2:**
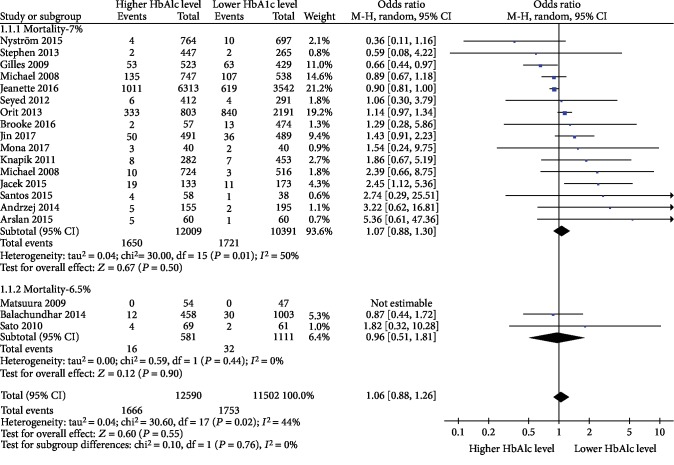
Impact of lower preoperative HbA1c levels and higher preoperative HbA1c levels on the incidence of mortality in diabetic patients after cardiac surgery.

For diabetic patients, 19 studies [23, 24, 26–35, 38–43, 45] reported on the incidence of mortality. In view of the different levels of HbA1c in different studies, the studies could not be pooled directly. We pooled the data using 6.5% and 7% as the lower and higher boundaries, respectively. The results of the meta-analysis showed that there was no significant difference in mortality (OR = 0.96, 95% CI 0.51-1.81, *P* = 0.90, and *I*^2^ = 0%) between diabetic patients with lower preoperative HbA1c levels (≤6.5%) and those with higher preoperative HbA1c levels (>6.5%) after cardiac surgery. No significant difference in mortality existed between diabetic patients with lower preoperative HbA1c levels (≤7%) and those with higher preoperative HbA1c levels (>7%) after cardiac surgery (OR = 1.07, 95% CI 0.88-1.30, *P* = 0.50, and *I*^2^ = 50%). Pooled estimates suggested no significant difference association of preoperative HbA1c level and mortality (OR = 1.06, 95% CI 0.88-1.26, *P* = 0.76, *I*^2^ = 0%) (Figure 2).

### 3.5. Infections of Surgical Sites

**Figure 3:**
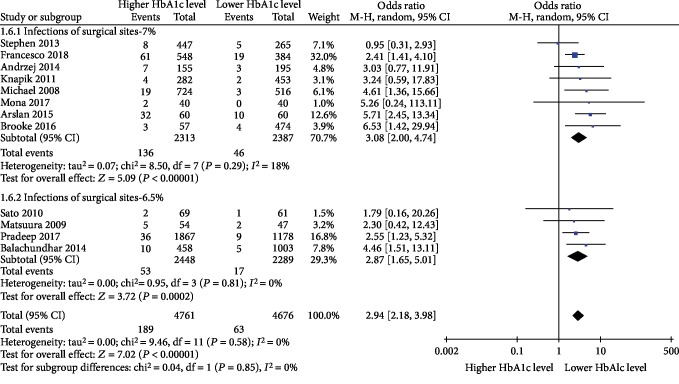
Impact of lower preoperative HbA1c levels and higher preoperative HbA1c levels on the incidence of surgical site infection in diabetic patients after cardiac surgery.

Twelve studies [23, 24, 26, 28, 31, 35–37, 39, 41–43] reported on the incidence of surgical site infection. The results of the meta-analysis showed that there was a strong association between surgical site infection rate and higher preoperative HbA1c levels in diabetic patients after cardiac surgery (OR = 2.94, 95% CI 2.18-3.98, *P* = 0.58, and *I*^2^ = 0%), as shown in Figure 3.

### 3.6. Stroke

**Figure 4:**
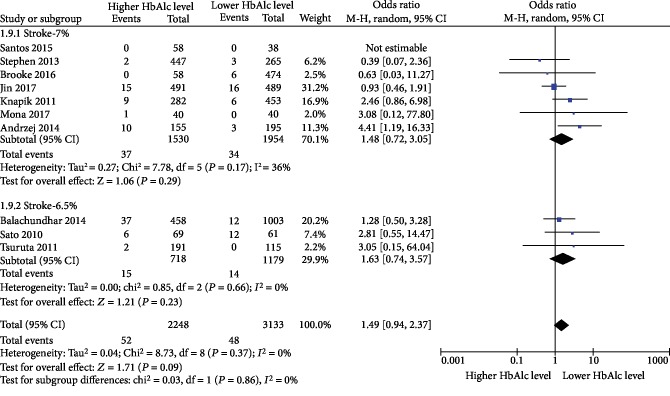
Impact of lower preoperative HbA1c levels and higher preoperative HbA1c levels on the incidence of stroke in diabetic patients after cardiac surgery.

Ten studies reported on the incidence of stroke [24, 26, 29, 31, 39–44]. The results of the meta-analysis showed that there was no significant difference in stroke incidence between diabetic patients with lower preoperative HbA1c levels and those with higher preoperative HbA1c levels after cardiac surgery (OR = 1.49, 95% CI 0.94-2.37, *P* = 0.37, and *I*^2^ = 8%), as shown in Figure 4.

### 3.7. Renal Failure

Nine studies reported on the incidence of renal failure [24, 28, 31, 35, 39, 40, 42–44]. The results of the meta-analysis suggested that a higher preoperative HbA1c level was associated with a high risk of renal failure after cardiac surgery (OR = 1.63, 95% CI 1.13-2.33, *P* = 0.63, and *I*^2^ = 0%) ([Supplementary-material supplementary-material-1]).

### 3.8. Myocardial Infarction

Nine studies reported on the incidence of myocardial infarction [24, 28–31, 34, 39, 40, 42]. The meta-analysis results showed that the incidence of myocardial infarction in diabetic patients after cardiac surgery was lower in the group with a lower preoperative HbA1c level than in the group with a higher preoperative HbA1c level (OR = 1.69, 95% CI 1.16-2.47, *P* = 0.47, and *I*^2^ = 0%), as shown in [Supplementary-material supplementary-material-1].

### 3.9. Hospital Stay

For diabetic patients, six studies reported on hospital stay [23, 26, 28, 35, 39, 41]. The results of the meta-analysis showed that a higher preoperative HbA1c level resulted in a 1.08-day mean increase in hospital stay after cardiac surgery (MD = 1.08, 95% CI 0.46-1.71, *P* = 0.28, and *I*^2^ = 21%), as shown in [Supplementary-material supplementary-material-1].

### 3.10. Atrial Fibrillation

Five studies reported on the incidence of atrial fibrillation [23, 28, 35, 39, 43]. The results of the meta-analysis showed that there was no significant difference in the incidence of atrial fibrillation between diabetic patients with lower preoperative HbA1c levels and those with higher preoperative HbA1c levels after cardiac surgery (OR = 0.94, 95% CI 0.67-1.33, *P* = 0.06, and *I*^2^ = 56%), as shown in [Supplementary-material supplementary-material-1].

### 3.11. Length of ICU Stay

Four studies reported on the length of time in the intensive care unit (ICU days) [23, 26, 31, 41]. The results of the meta-analysis showed that there was no significant difference in ICU days between diabetic patients with lower preoperative HbA1c levels and those with higher HbA1c levels after cardiac surgery (MD = 0.20, 95% CI -0.14-0.55, *P* = 0.40, and *I*^2^ = 0%), as shown in [Supplementary-material supplementary-material-1].

### 3.12. Sepsis

Four studies reported on the incidence of sepsis [26, 31, 40, 41]. The results of the meta-analysis showed that there was no significant difference in the incidence of sepsis between diabetic patients with lower preoperative HbA1c levels and those with higher HbA1c levels after cardiac surgery (OR = 2.49, 95% CI 0.99-6.25, *P* = 0.77, and *I*^2^ = 0%), as shown in [Supplementary-material supplementary-material-1].

### 3.13. Outcomes for Nondiabetic Patients

For nondiabetic patients, four studies were included [25, 28, 36, 37]: three studies included diabetic patients and nondiabetic patients [28, 36, 37] and one study included only diabetic patients [25]. The meta-analysis results are shown in Table 3.

### 3.14. Subgroup and Sensitivity Analysis

**Figure 5:**
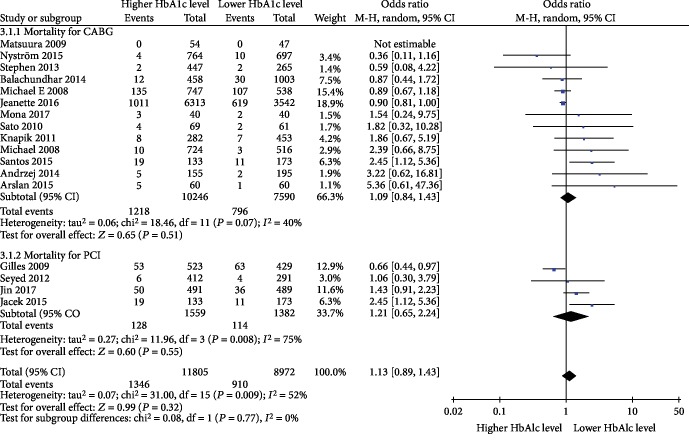
Subgroup analysis based on different types of cardiac surgery.

**Figure 6:**
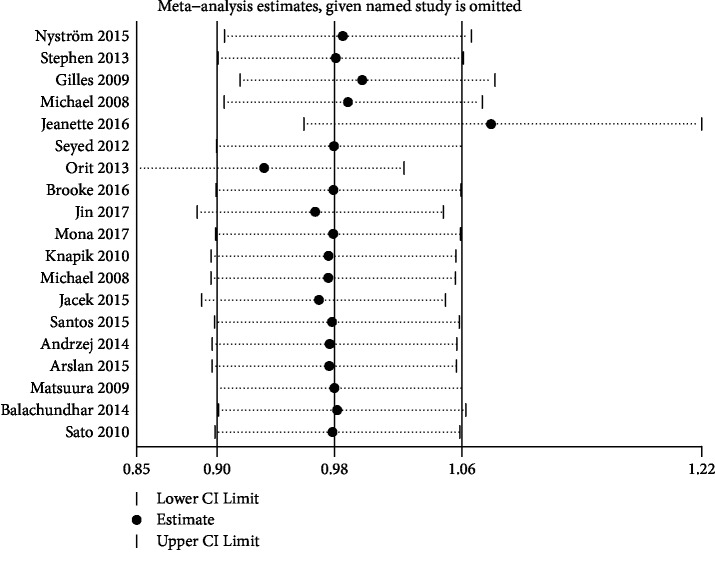
Sensitivity analysis for mortality in diabetic patients.

Based on different types of surgery (CABG and PCI), we performed subgroup analyses showing that mortality among those with different preoperative HbA1c levels did not reduce or increase in diabetic patients undergoing CABG (OR = 1.09, 95% CI 0.84-1.43, *P* = 0.07, and *I*^2^ = 40%) or PCI (OR = 1.21, 95% CI 0.65-2.24, *P* = 0.55, and *I*^2^ = 75%), as shown in Figure 5. Furthermore, we performed sensitivity analyses with the use of a metaninf command from Stata software for mortality in diabetic patients, and the results were not significantly different from those of the primary analysis, as shown in Figure 6.

### 3.15. Publication Bias

**Figure 7:**
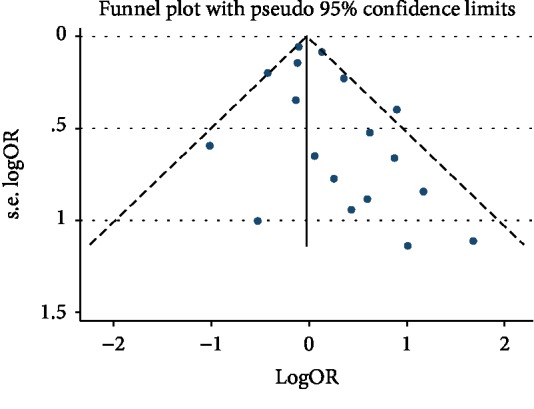
Funnel plots for mortality in diabetic patients.

Publication bias was tested by a visual examination of the funnel plots, which were symmetrical and showed no evidence of publication bias for mortality outcome, as shown in Figure 7.

### 3.16. Grading of Quality of Evidence

Table 4 details the quality of evidence for nine outcomes in the systematic review.

## 4. Discussion

This systematic review compared the association between the preoperative HbA1c levels and health outcomes of diabetic and nondiabetic patients who underwent cardiac surgery. The results showed that there were fewer surgical site infection events, fewer renal failure events, fewer myocardial infarction events, and shorter hospital stay times after cardiac surgery in diabetic patients with lower preoperative HbA1c levels (≤6.5% or 7%), and the quality of evidence was low to moderate. However, no significant difference was found between higher and lower preoperative HbA1c levels in the incidence of mortality (the quality of evidence was moderate) or stroke (the quality of evidence was moderate), the length of ICU stay (the quality of evidence was moderate), or the incidence of sepsis (the quality of evidence was low) or atrial fibrillation (the quality of the evidence was low) for diabetic patients. For nondiabetic patients who underwent cardiac surgery, we found a high risk of mortality and renal failure for patients with a higher preoperative HbA1c level, but in terms of myocardial infarction and atrial fibrillation, there was no significant difference between the patients with higher and lower preoperative HbA1c levels. Limited by the number of included studies, we did not perform a meta-analysis on other outcomes.

A current systematic review showed that perioperative hyperglycemia was not beneficial for patients undergoing cardiac surgery [7]. However, practitioners mainly focused on the relationship between preoperative blood glucose levels and the outcomes of cardiac surgery in patients with diabetes. A systematic review published in 2015 indicated that it was not necessary to measure preoperative blood glucose and HbA1c if the patients who underwent noncardiac surgery were without obvious clinical symptoms or signs of high risk [46]. Another systematic review [13] in the same year also obtained similar results. In addition, many systematic reviews that addressed the association between the preoperative HbA1c level and the outcomes of cardiac surgery in patients with diabetes have been published in the last [13, 47–49] years that showed that an elevated preoperative HbA1c level did not increase the postoperative morbidity and mortality in diabetic patients who underwent cardiac surgery. The results of our review were consistent with those of current systematic reviews, namely, the preoperative HbA1c level (we regarded 6.5% and 7% as the lower and higher boundaries, respectively) had no statistically significant effect on the incidence of mortality in diabetic patients after cardiac surgery. However, a systematic review published in 2017 [50] showed contrasting results, mainly because the authors did not include the PCI studies and nondiabetic patients. Our results might be influenced by the different definitions of higher and lower HbA1c levels in the included original studies. For nondiabetic patients, the results of this systematic review were consistent with those of a previous systematic review [51].

For the incidence rates of other complications, our systematic review found that a lower preoperative HbA1c level could reduce the incidence of surgical site infection, renal failure, and myocardial infarction, reducing the hospital stay in patients with diabetes mellitus after cardiac surgery. Several possible mechanisms may explain the results. HbA1c is an indicator of long-term (3- to 4-month) glycemic control and is formed when glucose in the blood binds irreversibly to hemoglobin to form a stable glycated hemoglobin complex. HbA1c is not affected by acute changes in blood glucose levels [52]. In patients with type 2 diabetes, HbA1c > 7% can induce adverse metabolic memory. The higher the HbA1c, the higher the risk of macrovascular complications, microvascular complications, and death in diabetic patients [53]. HbA1c induces dyslipidemia, hyperhomocysteinemia, and hypertension and increases C-reactive protein, oxidative stress, and blood viscosity, which would contribute to the development of cardiovascular diseases [54]. Surgery, stress, and anesthesia can exacerbate oxidative stress and increase blood viscosity. For diabetic patients, the situation worsens [55]. In the case of stress and blood loss, to protect the blood supply of important organs, visceral large blood vessels contract. The kidney is one of the most sensitive organs for blood volume. When the blood volume is insufficient, kidney failure can occur at the earliest. Patients with long-term hyperglycemia due to osmotic diuresis may experience a relative lack of blood volume. Insufficient blood volume during the perioperative period is more obvious and may be one of the important causes of renal failure [56]. Increased blood viscosity is one of the important factors leading to the occurrence of large blood clots. The high blood viscosity of diabetic patients can induce myocardial infarction [57]. Decreased wound healing ability, weakened inflammatory cell chemotaxis, and decreased immune function in diabetic patients are the main causes of postoperative infection. High HbA1c can lead to lower wound healing, weakened chemokine chemotaxis, and decreased immune function in diabetic patients, which can increase infection [58]. For these reasons, high HbA1c levels will prolong the hospital stay in patients with diabetes.

This systematic review had the following strengths: (1) To our knowledge, this was the first designed systematic review to investigate the effect of the preoperative HbA1c level on the postoperative outcomes in diabetic and nondiabetic patients who underwent cardiac surgery; the current systematic review mainly focused on coronary artery disease or percutaneous coronary interventions. (2) Our study is registered in PROSPERO, which could improve the overall reporting and methodological quality [59]. (3) We conducted a quality assessment using the GRADE system for each outcome compared with a previous systematic review. The following limitations should be taken into consideration: (1) We included cohort studies that had a potential high risk of bias. (2) The definitions of higher preoperative HbA1c levels were different in the original studies and included multiple values, such as 6.5%, 7%, 7.5%, and 8%. Although we grouped them for analysis, the grouping might not be very accurate and may have caused heterogeneity. (3) The follow-up time for postoperative mortality varied greatly (ranging from before discharge to 7 years after surgery). (4) The postoperative glycemic index is probably a confounding factor that affects the correlation of events after cardiac surgery with HbA1c levels.

## 5. Conclusions

The results of this systematic review showed that patients with diabetes mellitus with lower preoperative HbA1c levels showed a reduced incidence of surgical site infection, renal failure, and myocardial infarction, as well as a reduced length of hospital stay, after cardiac surgery compared with those with higher HbA1c levels, and the quality of evidence was low to moderate. However, the higher level of preoperative HbA1c had no effect on the incidence of mortality or other adverse events. Our results were based on current available evidence, and the conclusions can provide guidance for clinical practice to some extent. However, there was great inconsistency in the definition of higher preoperative HbA1c levels in the included studies, and we still need high-quality RCTs with large sample sizes to further investigate this issue in the future.

## Figures and Tables

**Table 1:** Baseline characteristics of the included studies.

No.	Study design	Type of cardiac surgery	Type of diabetes	Follow-up time	Sample size	Number of patients in exposure group	Number of patients in controlled group	Preoperative HbA1c level in exposure group (%)	Preoperative HbA1c level in controlled group (%)
Nicolini et al. 2018 [37]	Cohort study	CABG	DM	NR	942	384	548	<7.0	≥7.0
Narayan et al. 2017 [36]	Retrospective study	CABG	DM	NR	3045	1178	1867	<6.5	≥6.5
Ramadan et al. 2017 [39]	Cohort study	CABG	T1DM, T2DM	1 y	80	40	40	≤7.0	>7.0
Hwang et al. 2017 [29]	Cohort study	PCI	T2DM	5.4 y	980	489	491	<7.0	≥7.0
Kuhl et al. 2016 [33]	Cohort study	CABG	T2DM	5.5 ± 3.8 y	6313	2771	3542	≤7.0	>7.0
Finger et al. 2016 [26]	Cohort study	CABG	DM	NR	531	474	57	≤7.0	>7.0
Ümit et al. 2015 [23]	Cohort study	CABG	DM	NR	120	60	60	<7.0	≥7.0
Santos et al. 2015 [40]	Prospective study	CABG	T1DM	2 y	96	38	58	≤7.0	>7.0
Kowalczyk et al. 2015 [32]	Cohort study	PCI	New DM	2 y	306	173	133	≤7.0	>7.0
Nystrom et al. 2015 [38]	Cohort study	CABG	T1DM	4.7 y	766	67	697	≤7.0	>7.0
Subramaniam et al. 2014 [43]	Cohort study	CABG	DM	NR	1461	1003	458	<6.5	≥6.5
Biskupski et al. 2014 [24]	Cohort study	CABG	T2DM	NR	350	195	155	<7.0	≥7.0
Twito et al. 2013 [45]	Cohort study	CABG, PCI	New DM	7 y	2994	2191	803	<7.0	≥7.0
Strahan et al. 2013 [42]	Prospective study	CABG	DM	NR	712	265	447	<7.0	≥7.0
Kassaian et al. 2012 [30]	Cohort study	PCI	DM	1 y	703	291	412	≤7.0	>7.0
Tsuruta et al. 2011 [44]	Cohort study	CABG	DM	3.6 ± 1.7 y	306	115	191	<6.5	≥6.5
Knapik et al. 2011 [31]	Cohort study	CABG	DM	NR	735	453	282	≤7.0	>7.0
Sato et al. 2010 [41]	Cohort study	CABG	T2DM	30 days	130	61	69	<6.5	>6.5
Matsuura et al. 2009 [35]	Retrospective study	CABG	DM	2.4 ± 1.6 y	101	47	54	<6.5	>6.5
Lemesle et al. 2009 [34]	Cohort study	PCI	DM	1 y	952	429	523	≤7.0	>7.0
Halkos et al. 2008 [27]	Cohort study	CABG	DM	5 y	1285	538	747	<7.0	≥7.0
Halkos et al. 2008 [28]	Cohort study	CABG	DM	NR	1240	516	724	<7.0	≥7.0
Nicolini et al. 2018 [37]	Cohort study	CABG	Non-DM	NR	1664	1519	145	<7.0	≥7.0
Narayan et al. 2017 [36]	Retrospective study	CABG	Non-DM	NR	1633	1298	335	<6.5	≥6.5
El-sherbiny et al. 2015 [25]	Prospective study	PCI	Non-DM	0.5 y	60	27	33	<6.5	≥6.5
Halkos et al. 2008 [28]	Cohort study	CABG	Non-DM	NR	1240	1759	90	<7.0	≥7.0

NR: not reported.

**Table 2:** Risk of bias for included studies.

No.	Included studies	①	②	③	④	⑤	⑥	⑦	⑧	Overall score
1	Jin 2017	1	0	1	1	1	1	0	0	5
2	Andrzej 2014	1	1	0	1	1	0	1	1	6
3	Balachundhar 2014	1	1	0	1	1	1	0	0	5
4	Brooke 2016	1	1	1	1	1	0	0	0	5
5	Francesco 2018	1	0	1	1	1	1	0	0	5
6	Jacek 2015	1	1	0	1	1	1	1	1	7
7	Jeanette 2016	1	0	1	1	1	0	1	1	6
8	Mona 2017	1	1	1	1	1	0	0	0	5
9	Orit 2013	1	1	0	1	1	1	0	0	5
10	Sato 2010	1	1	1	1	1	0	0	0	5
11	Seyed 2012	1	1	1	1	1	1	1	1	8
12	Michael 2008	1	1	1	1	1	0	1	1	7
13	Santos 2015	1	1	1	1	1	0	0	0	5
14	Gilles 2009	1	1	1	1	1	0	1	1	7
15	Nyström 2015	1	1	1	1	1	0	1	1	7
16	Tsuruta 2011	1	1	1	1	1	0	1	1	7
17	Knapik 2010	1	1	1	1	2	0	0	0	6
18	Matsuura 2009	1	1	1	1	1	0	1	1	7
19	Arslan 2015	1	0	1	1	1	1	0	0	5
20	Pradeep 2017	1	1	1	1	1	1	0	0	6
21	Stephen 2013	1	1	1	1	1	1	1	0	7
22	Michael 2008	1	1	1	1	1	0	0	0	5
23	Islam 2015	1	1	1	1	1	1	1	0	7

^①^Representativeness of the exposed cohort. ^②^Selection of the nonexposed cohort. ^③^Ascertainment of exposure. ^④^Demonstration that the outcome of interest was not present at the start of the study. ^⑤^Comparability of cohorts on the basis of the design or analysis. ^⑥^Assessment of outcome. ^⑦^Follow-up was long enough for outcomes to occur. ^⑧^Adequacy of follow-up of cohorts.

**Table 3:** Meta-analysis of health-related outcomes according to preoperative HbA1c level after cardiac surgery for nondiabetic patients.

Outcome	Studies	Higher HbA1c level	Lower HbA1c level	Statistical method	Effect estimate
Mortality	3 [25, 28, 36]	458	3084	OR, random	2.23 [1.01, 4.90]^∗^
Myocardial infarction	2 [25, 28]	123	1786	OR, random	1.32 [0.27, 6.31]
Atrial fibrillation	2 [28, 36]	425	3057	OR, random	0.99 [0.74, 1.33]
Renal failure	2 [28, 36]	425	3057	OR, random	2.33 [1.32, 4.12]^#^

^∗^ *P* = 0.03; ^#^*P* = 0.004.

**Table 4:** Quality of evidence of ten outcomes in diabetic patients after cardiac surgery.

Quality assessment								Effect size	Quality of the evidence (GRADE)
No. of studies (sample size)	Study design	Risk of bias	Inconsistency	Indirectness	Imprecision	Publication bias	Rating up factor		
Mortality: Higher HbA1c level vs lower HbA1c level (preoperative)									
19 (24092)	Cohort study	Serious^a^	Not serious	Not serious	Not serious	Undetected	No	OR 1.06 [0.88, 1.26]	⊕⊕⊕Ο
									Moderate
Surgical site infection: higher HbA1c level vs. lower HbA1c level (preoperative)									
12 (9437)	Cohort study	Serious^a^	Not serious	Not serious	Not serious	Undetected	No	OR 2.94 [2.18, 3.98]	⊕⊕⊕Ο
									Moderate
Stroke: higher HbA1c level vs. lower HbA1c level (preoperative)									
10 (5381)	Cohort study	Serious^a^	Not serious	Not serious	Not serious	Undetected	No	OR 1.49 [0.94, 2.37]	⊕⊕⊕Ο
									Moderate
Renal failure: higher HbA1c level vs lower HbA1c level (preoperative)									
9 (5081)	Cohort study	Serious^a^	Serious^b^	Not serious	Not serious	Undetected	No	OR 1.63 [1.13, 2.33]	⊕⊕ΟΟ
									Low
Myocardial infarction: higher HbA1c level vs. lower HbA1c level (preoperative)									
9 (5848)	Cohort study	Serious^a^	Not serious	Not serious	Not serious	Undetected	No	OR 1.69 [1.16, 2.47]	⊕⊕⊕Ο
									Moderate
Hospital stay: higher HbA1c level vs. lower HbA1c level (preoperative)									
6 (2202)	Cohort study	Serious^a^	Serious^b^	Not serious	Not serious	Undetected	No	MD 1.08 [0.46, 1.71]	⊕⊕ΟΟ
									Low
Atrial fibrillation: higher HbA1c level vs. lower HbA1c level (preoperative)									
5 (3002)	Cohort study	Serious^a^	Serious^b^	Not serious	Not serious	Undetected	No	OR 0.94 [0.67, 1.33]	⊕⊕ΟΟ
									Low
Length of ICU stay: higher HbA1c level vs. lower HbA1c level (preoperative)									
4 (1121)	Cohort study	Serious^a^	Not serious	Not serious	Not serious	Undetected	No	OR 0.20 [-0.14, 0.55]	⊕⊕⊕Ο
									Moderate
Sepsis: higher HbA1c level vs. lower HbA1c level (preoperative)									
4 (1492)	Cohort study	Serious^a^	Not serious	Not serious	Serious^c^	Undetected	No	OR 2.49 [0.99, 6.25]	⊕⊕ΟΟ
									Low

High: we are very confident that the true effect lies close to that of the estimate of the effect; moderate: we are moderately confident in the effect estimate—the true effect is likely to be close to the estimate of the effect, but there is a possibility that it is substantially different; low: our confidence in the effect estimate is limited—the true effect may be substantially different from the estimate of the effect; very low: we have very little confidence in the effect estimate—the true effect is likely to be substantially different from the estimate of effect. ^a^The poor quality of included studies in the independent blind assessment of outcomes and inadequate follow-up time. ^b^Serious inconsistency for the scattered 95% CI. ^c^Wide confidence intervals, serious imprecision.

## Abbreviations

HbA1c: Glycosylated hemoglobin A1c
DM: Diabetes mellitus
ICU: Intensive care unit
SR: Systematic review
CNKI: China National Knowledge Infrastructure
CBM: Chinese Biology Medicine
ICPTR: International Clinical Trials Registry Platform
NHS: National Health Service.

## Supplementary Materials

Supplementary 1 Annex 1: PRISMA checklist.

Supplementary 2 Annex 2: search strategy.

Supplementary 3 Annex 3: meta-analysis of secondary outcomes for diabetic patients after cardiac surgery.
