# Supplementary material for: Effects of Preoperative HbA1c Levels on the Postoperative Outcomes of Coronary Artery Disease Surgical Treatment in Patients with Diabetes Mellitus and Nondiabetic Patients: A Systematic Review and Meta-Analysis
Source: J Diabetes Res. 2020 Feb 28;2020:3547491. doi: 10.1155/2020/3547491 (PMC7066407; doi:10.1155/2020/3547491)
Supplement: Supplementary 2 — Annex 2: search strategy. [file 3547491.f2.docx]

**Annex1 Search Strategy**

**PubMed（191）**

1. ‘Coronary Artery Bypass’[Mesh]
2. ‘Thoracic surgical procedures’[Title/Abstract]
3. ‘Thoracic Surgery’[Title/Abstract]
4. ‘Aortocoronary Bypass’[Title/Abstract]
5. ‘Cardiac surgery’[Title/Abstract]
6. ‘Cardiac surgical procedures’[Title/Abstract]
7. ‘Cardiovascular Surgical Procedure’[Title/Abstract]
8. CABG[Title/Abstract]
9. ‘coronary surgery’[Title/Abstract]
10. ‘coronary artery bypass’[Title/Abstract]
11. ‘heart surgery’[Title/Abstract]
12. ‘Heart Surgical Procedure’[Title/Abstract]
13. OR/#1-#12
14. glycosylated hemoglobin[Title/Abstract]
15. Glycated Hemoglobin[Title/Abstract]
16. HbA1c[Title/Abstract]
17. "Glycated Hemoglobin A"[Mesh]
18. "hemoglobin A1c protein, human" [Supplementary Concept]
19. "pre-hemoglobin A, glycosylated" [Supplementary Concept]
20. OR/#14-#19
21. #13 AND #20

**TheCochraneLibrary（77）**

1. Coronary Artery Bypass:ti,ab,kw
2. Thoracic surgical procedures:ti,ab,kw
3. Thoracic Surgery:ti,ab,kw
4. Aortocoronary Bypass:ti,ab,kw
5. Cardiac surgery:ti,ab,kw
6. Cardiac surgical procedures:ti,ab,kw
7. Cardiovascular Surgical Procedure:ti,ab,kw
8. CABG:ti,ab,kw
9. coronary surgery:ti,ab,kw
10. heart surgery:ti,ab,kw
11. Heart Surgical Procedure:ti,ab,kw
12. MeSH descriptor: [Coronary Artery Bypass] explode all trees
13. MeSH descriptor: [Thoracic Surgery] explode all trees
14. OR/#1-#13
15. glycosylated hemoglobin:ti,ab,kw
16. Glycated Hemoglobin:ti,ab,kw
17. HbA1c:ti,ab,kw
18. MeSH descriptor: [Glycated Hemoglobin A] explode all trees
19. OR/#15-#18
20. #14 AND #19

**WOS（236）**

1. TOPIC: (Coronary Artery Bypass)
2. TOPIC: (Thoracic surgical procedures)
3. TOPIC: (Thoracic Surgery)
4. TOPIC: (Aortocoronary Bypass)
5. TOPIC: (Cardiac surgery)
6. TOPIC: (Cardiac surgical procedures)
7. TOPIC: (Cardiovascular Surgical Procedure)
8. TOPIC: (CABG)
9. TOPIC: (coronary surgery)
10. TOPIC: (heart surgery)
11. TOPIC: (Heart Surgical Procedure)
12. OR/#1-#11
13. TOPIC：(glycosylated hemoglobin)
14. TOPIC: (Glycated Hemoglobin)
15. TOPIC: (HbA1c)
16. OR/#13-#15
17. #12 AND #16

**Embase（382）**

1. 'coronary artery bypass':ab,ti
2. 'thoracic surgical procedures':ab,ti
3. 'thoracic surgery':ab,ti
4. 'aortocoronary bypass':ab,ti
5. 'cardiac surgery':ab,ti
6. 'cardiac surgical procedures':ab,ti
7. 'cardiovascular surgical procedure':ab,ti
8. 'cabg':ab,ti
9. 'coronary surgery':ab,ti
10. 'heart surgery':ab,ti
11. 'heart surgical procedure':ab,ti
12. 'coronary artery bypass graft'/exp
13. 'thorax surgery'/exp
14. 'heart surgery'/exp
15. OR/#1-#14
16. 'glycosylated hemoglobin':ab,ti
17. 'glycated hemoglobin':ab,ti
18. 'hba1c':ab,ti
19. OR/#16-#18
20. #15 AND #19
21. #20 AND [medline]/lim
22. #20 NOT #21
