# Supplementary material for: Effects of Preoperative HbA1c Levels on the Postoperative Outcomes of Coronary Artery Disease Surgical Treatment in Patients with Diabetes Mellitus and Nondiabetic Patients: A Systematic Review and Meta-Analysis
Source: J Diabetes Res. 2020 Feb 28;2020:3547491. doi: 10.1155/2020/3547491 (PMC7066407; doi:10.1155/2020/3547491)
Supplement: Supplementary 3 — Annex 3: meta-analysis of secondary outcomes for diabetic patients after cardiac surgery. [file 3547491.f3.docx]

Annex 3Meta-analysisofSecondoutcomesfordiabetic patients after cardiac surgery.


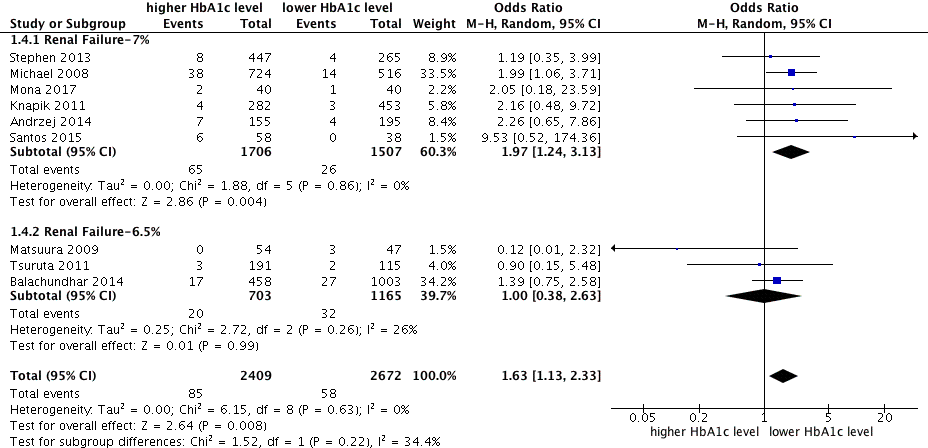


**A****nnex 3-1** Impact of preoperative lower HbA1c level and higher HbA1c level on renal failure in diabetic patients after cardiac surgery


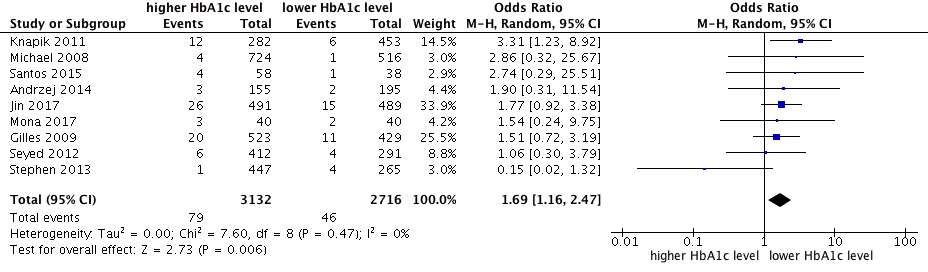


**Annex 3-2** Impact of preoperative lower HbA1c level and higher HbA1c level on myocardial infarction in diabetic patients after cardiac surgery


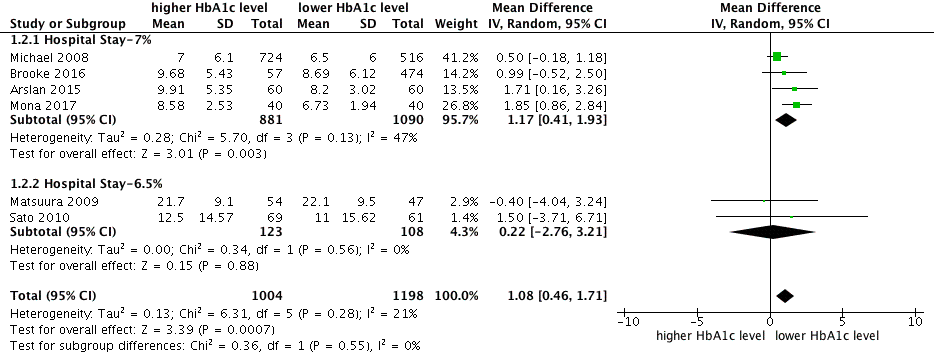


**Annex 3-3** Impact of preoperative lower HbA1c level and higher HbA1c level on hospital stay in diabetic patients after cardiac surgery


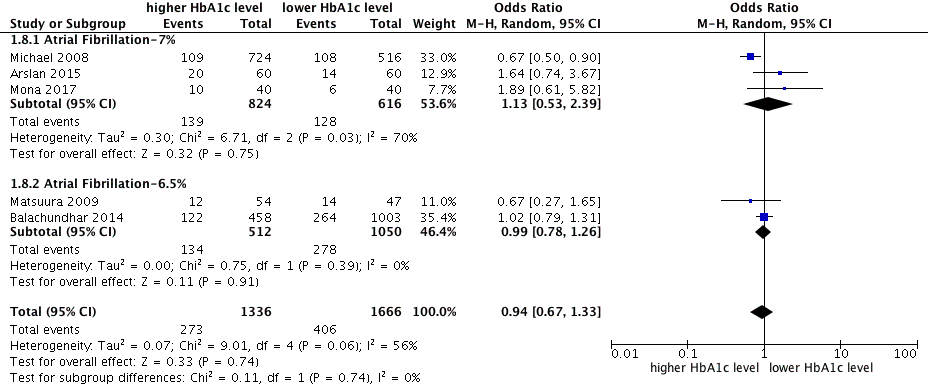


**Annex 3-4** Impact of preoperative lower HbA1c level and higher HbA1c level on atrial fibrillation in diabetic patients after cardiac surgery


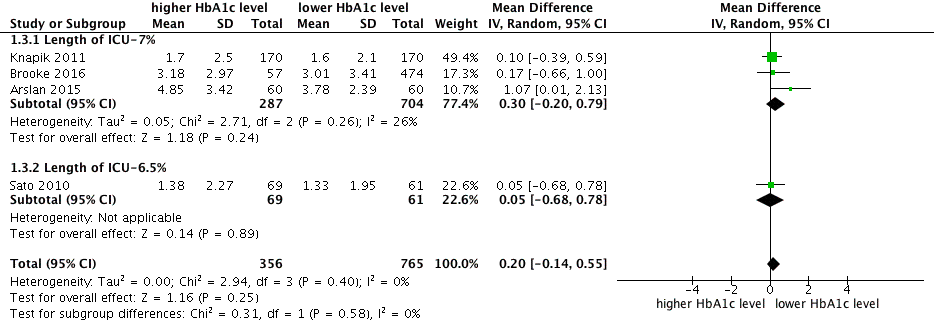


**Annex 3-5** Impact of preoperative lower HbA1c level and higher HbA1c level on ICU days in diabetic patients after cardiac surgery


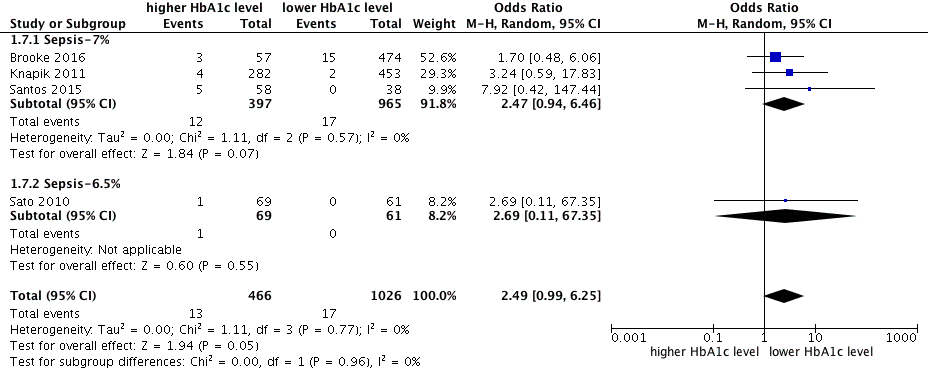


**Annex 3-6** Impact of preoperative lower HbA1c level and higher HbA1c level on sepsis in diabetic patients after cardiac surgery
